# A Comprehensive 10-Year Nationwide Pharmacovigilance Surveillance on Antibacterial Agents in Korea: Data Mining for Signal Detection of Trends and Seriousness of Adverse Events

**DOI:** 10.3390/microorganisms13010136

**Published:** 2025-01-10

**Authors:** Seon Hu Mo, Soo Hyeon Lee, Chang-Young Choi, Yongjun Sunwoo, Sooyoung Shin, Yeo Jin Choi

**Affiliations:** 1 Department of Pharmaceutical Science, College of Pharmacy, Kyung Hee University, Seoul 02447, Republic of Korea; 2 Department of Regulatory Science, Graduate School, Kyung Hee University, Seoul 02447, Republic of Korea; 3 Institute of Regulatory Innovation through Science (IRIS), Kyung Hee University, Seoul 02447, Republic of Korea; 4 Department of Gastroenterology, Korea Medical Institute, Suwon 16553, Republic of Korea; 5 Department of Pharmacy, College of Pharmacy, Ajou University, Suwon 16499, Republic of Korea; 6 Research Institute of Pharmaceutical Science and Technology (RIPST), Ajou University, Suwon 16499, Republic of Korea; 7 Department of Pharmacy, College of Pharmacy, Kyung Hee University, Seoul 02447, Republic of Korea

**Keywords:** bacterial infection, drug safety, antibacterial, real-world data, pharmacovigilance

## Abstract

A comprehensive pharmacovigilance surveillance on antibacterials is lacking. This study aims to investigate safety signals of antibacterial-related adverse drug events (ADEs) with seriousness and to identify predictors of serious ADEs. This study investigated 52,503 antibacterial-induced ADEs reported to the Korea Adverse Event Reporting System Database from January 2013 to December 2022. Disproportionality analysis was conducted, and the effect sizes were estimated by reporting odds ratios (ROR), proportional reporting ratio (PRR), and information component (IC). Multivariate logistic regression was performed to investigate the predictors of serious ADEs by estimating the odds ratio (OR). Serious events were more likely to be cardiovascular disorders (ROR 6.77, PRR 6.6, IC 2.37), urinary system disorders (ROR 5.56, PRR 5.22, IC 2.12), and platelet, bleeding, and clotting disorders (ROR 5.41, PRR 5.17, IC 2.06). The predictors may include age (OR 1.05), the number of concomitant medications (OR 1.44), concomitant proton pump inhibitors (OR 1.46) and non-steroidal anti-inflammatory drugs (OR 1.38) use, and specific antibacterial classes, while multiple antibacterial therapy was associated with lower serious ADE risks. The sensitivity analysis also suggests the male sex (OR 1.18) as a potential predictor of serious ADEs. However, further studies are imperative to determine the causality of antibacterial-induced ADEs in critically ill patients.

## 1. Introduction

Antibacterial agents are essential for treating bacterial infections, and their appropriate use is imperative for optimal infection control as well as antibacterial resistance prevention [1]. Global antibacterial use has surged in recent decades, with an estimated global increase of 46% between 2000 and 2018 [2]. Although the studies suggested declined antibacterial consumption at the start of COVID-19, antibacterial sales for major antibiotics, including penicillin, cephalosporins, and macrolides, quickly rebounded and were positively associated with rising global COVID-19 cases during the pandemic period [3]. The incidence of antibacterial resistance has been increasing over the past decades, driven by longstanding overuse and misuse of antibiotics [4]. While the COVID-19 pandemic brought renewed attention to infection control, the emergence of multidrug-resistant (MDR) or extensively drug-resistant (XDR) pathogens, which preceded the pandemic, still remains an intensifying global health concern, and the prevalence of infections from MDR or XDR pathogens is worsening, accentuating increasing challenges to public health systems worldwide [5].

The antimicrobial stewardship program (ASP) has been endorsed in the healthcare system, especially within institutions, to guide and reinforce judicious antibacterial use. ASPs are designed to promote appropriate antibacterial use, ultimately protecting patients from harm and antibacterial resistance by unnecessary antibiotic use [6]. Numerous studies, including meta-analyses, indeed have revealed a substantial decline in antibacterial prescription and consumption with ASP practice [6]. However, optimal ASP should not only engage in the selection of appropriate antibacterials that match the spectrum of activity but also in minimizing the adverse drug events (ADEs) associated with antibacterial use. Uncontrolled infection certainly endangers patients, as implied by 13.7 million infection-related deaths in 2019 [7], and antibiotic resistance assuredly contributes to substantial mortality, responsible for 4.71 million deaths in 2021 [4]. Meanwhile, mortality or morbidity associated with antibiotic-induced ADEs is easily overlooked in clinical practice, and effective stewardship should also focus on minimizing ADEs that can arise from antibacterial therapy.

While the majority of ADEs are mild and self-limiting, severe ADEs can lead to life-threatening reactions, including anaphylaxis and organ toxicity, which may result in prolonged hospitalization or even death [8]. A recent cohort study emphasized the substantial impact of antibacterial-mediated anaphylactic reactions on mortality, estimating a mortality rate of 18.6 cases per 100,000 exposures [9]. Another cohort study suggested that almost 20% of hospitalized patients experience at least one antibiotic-associated ADE during their hospitalizations, and increases in treatment duration of antibiotics conferred a 3% ADE risk [10]. Furthermore, findings from our previous pharmacovigilance study on drug-induced fatal events identified antibacterials as the most frequent contributors to drug-induced fatalities, accounting for 20.3% of all fatal ADEs [8]. Despite these substantial risks, comprehensive pharmacovigilance studies on antibacterials are still limited [8]. Investigating the nature, prevalence, and predictors of antibacterial-associated ADEs is crucial for ensuring patient safety, especially in populations that may be more vulnerable to adverse outcomes. Hence, this study aims to characterize the prevalence and seriousness of antibacterial-related ADEs and to identify predictors that may increase the likelihood of serious ADEs, ultimately enhancing infection control and promoting patient safety and appropriate antibacterial use.

## 2. Materials and Methods

### 2.1. Data Source, Definition and Data Acquisition

This study was a cross-sectional study conducted in accordance with the Strengthening the Reporting of Observational Studies in Epidemiology (STROBE) guideline (Supplementary Table S1). This study analyzed spontaneous adverse events (AEs) records reported to a nationwide pharmacovigilance system named the Korean Adverse Event Reporting System Database (KIDS KAERS DB), constructed by the Korean Institute of Drug Safety and Risk Management (KIDS, Ministry of Food and Drug Safety) [11]. A causality assessment was conducted for all ADEs reported in the KIDS KAERS DB. The causality of each case was verified by healthcare professionals designated by the KIDS and confirmed through a review of scientific pharmacovigilance data provided by the manufacturers, patient medical charts, and interviews with patients and involved healthcare professionals to minimize potential bias [11].

Antibiotic-related AE cases reported from 1 January 2013 to 31 December 2022 were included in the analysis. All antibiotic-related ADE cases classified as “certain”, “probable/likely”, and “possible” causality per World Health Organization-Uppsala Monitoring Centre (WHO-UMC) criteria were analyzed [12]. All adverse drug events (ADE) types and reactions were reported in accordance with the Medicinal Dictionary for Regulatory Activities (MedDRA) and were further classified into system organ classes (SOC). Serious adverse events (SAEs) were defined as any antibiotic-induced AEs involved with (1) death, (2) life-threatening AEs, (3) hospitalization or prolonged existing hospitalizations, (4) persistent or significant incapacity or substantial disruption of the ability to conduct normal life functions, (5) congenital abnormality or birth defects, or (6) other medically significant events in accordance with the International Conference on Harmonization (ICH) E2D guideline [13].

The following information was extracted from KIDS KAERS DB for the analysis: (1) patient demographic information such as sex and age, (2) medical histories or comorbidities, (3) information pertaining to etiologic and concurrent medications, (4) ADE types reported with MedDRA, and (5) causality assessment along with the seriousness of AEs. Each event was analyzed as a separate case when two or more AEs were reported in the same patient. The study protocol utilizing AE records from KIDS KAERS DB was approved by the Korea Institute of Drug Safety and Risk Management (Ministry of Food and Drug Safety) (KIDS KAERS DB 2301A0005) and the institutional review board of Kyung Hee University (No. KHSIRB-23-435) (Seoul, Republic of Korea). Informed consents were exempted by the board.

### 2.2. Statistical Analysis

Patient demographics and ADE types related to antibiotic use were summarized using descriptive statistics. Continuous variables such as age were expressed as median (range) based on Kolmogorov–Smirnov normality test results. ADEs with at least four reported cases of both nonserious ADEs and SAEs were subjected to the disproportionality analysis to ensure the validity and reliability of the results [14,15]. Disproportionality analysis was performed to determine the association between antibiotic classes and the seriousness of ADEs, as well as the association of the SOC-based ADEs with their seriousness. The association between SOC-based ADEs and seriousness was further evaluated for each antibacterial class. The results of disproportionality analysis were estimated by reporting odds ratios (RORs) with the corresponding 95% confidence intervals (CIs) and the Mantel–Haenszel-adjusted *p*-values. Furthermore, the proportional reporting ratio (PRR) and the information component (IC) from the Bayesian Confidence Propagation Neural Network (BCPNN) were estimated for the identified ADEs to further evaluate the strength of the association between antibiotic classes and the seriousness of ADEs based on SOC [16]. IC > 0 indicates that the number of observed ADEs is greater than the expected number of ADEs, and the higher the IC value, the stronger the association [16]. The univariate logistic regression analysis was performed to identify factors associated with increased risk of SAEs, and the factors encompassed sex, age, number of concomitantly administered medications, number of concomitantly administered antibacterials, types of concomitantly administered medications, and causality (certain, probable/likely, or possible). Multiple logistic regression using the forward selection method was performed to investigate the factors that are substantially associated with increased risk of SAEs from the univariate analysis. Sensitivity analysis was performed to detect safety signals of antibacterials in critically ill patients. As the majority of patient medical history information was missing, we performed disproportionality and logistic regression on ADE cases related to antibacterials commonly used for treating MDR Gram-negative bacterial infections, which are frequently administered in critically ill patients [17]. The list of antibiotics prescribed for MDR Gram-negative bacteria infection was extracted from the clinical guideline, “Infectious Disease Society of America 2024 Guidance on Treatment of Antimicrobial-Resistant Gram-Negative Infections” [17]. All statistical analyses were performed using SPSS Statistics 26.0 (IBM SPSS Statistics for Windows, Armonk, NY, USA), with statistical significance set at *p*-values < 0.05. Heatmaps for visual representation of disproportionality analyses were generated using R (version 4.4.5).

## 3. Results

### 3.1. Baseline Demographics

**Figure 1:**
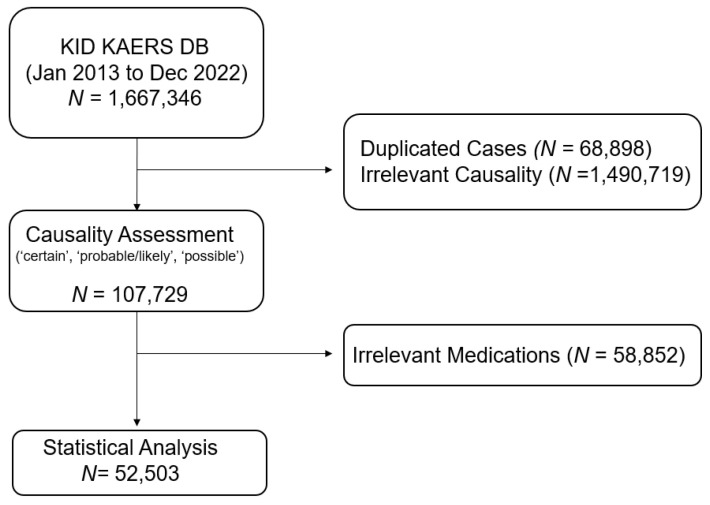
Data acquisition process of the study.

Among 1,667,346 cases obtained from KIDS KAERS DB, a total of 52,503 antibiotic-related ADE cases in 30,979 patients were included in the analysis based on the WHO-UMC causality assessment from 1 January 2013 to 31 December 2022 (Figure 1). The baseline demographic characteristics of the included ADE reports are summarized in Table 1. Approximately 40% of antibiotic-related ADEs were reported in patients aged 60 years and older (n = 20,902), and the majority of ADE cases were reported in patients aged 70 years and older (n = 12,131, 23.60%). The prevalence of serious antibiotic-induced ADEs was 5.60% (n = 2917). Almost 30% of patients administered at least two antibacterials concomitantly (n = 15,942). Cephalosporins (n = 18,184, 34.63%) were the most common etiologic antibiotic class, followed by fluoroquinolones (n = 9197, 17.52%) and penicillin (n = 7623, 14.52%).

### 3.2. Types of ADEs

**Figure 2:**
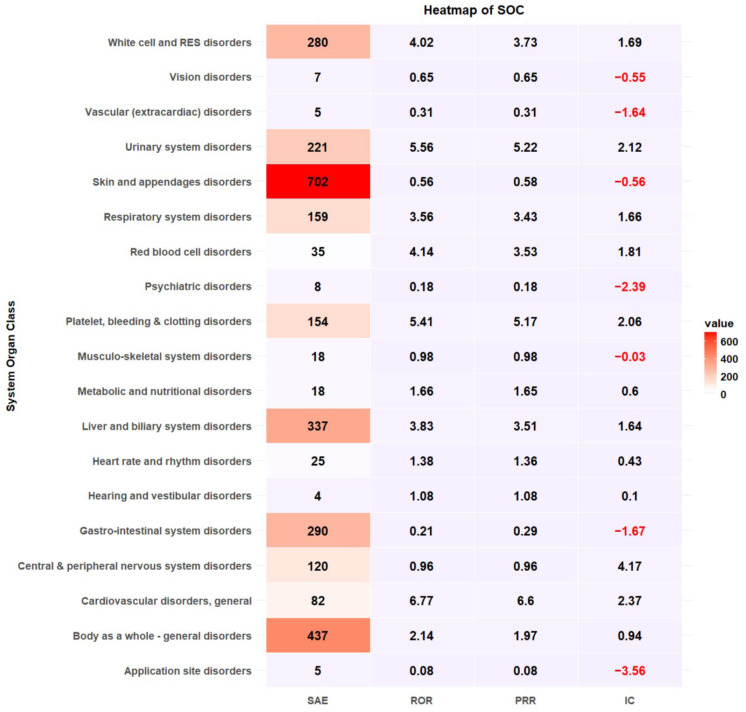
Heatmap of signal detection for antibacterial-associated ADEs.

Cephalosporins were responsible for the most reported SAEs (n = 902, 30.92%), followed by fluoroquinolones (n = 516, 17.69%) and penicillin (n = 406, 13.92%) (Table 2). Antibacterial classes, including glycopeptides (ROR 1.85, 95% CI 1.65–2.07), lincomycin (ROR 1.41, 95% CI 1.15–1.73), oxazolidinone (ROR 2.90, 95% CI 1.78–4.72), aminoglycosides (ROR 1.34, 95% CI 1.10–1.63), sulfamethoxazole/trimethoprim (ROR 3.66, 95% CI 2.80–4.78), and polymyxin B (ROR 3.83, 95% CI 1.85–7.91), were more likely to report SAEs (Table 2). The most common antibiotic-induced ADEs were skin and appendage disorders (n = 18,607, 35.44%) and gastrointestinal disorders (n = 17,417, 33.17%) (Table 3). The most prevalent SAEs involved skin and appendage disorders (n = 702, 24.06%), followed by the body as a whole-general disorder (n = 437, 14.98%) and liver and biliary system disorders (n = 337, 11.55%). The safety signal detection results of SOC-based ADEs and their seriousness are summarized and visualized in Table 3 and Figure 2, respectively. ADEs classified as liver and biliary system disorders (ROR 3.83, 95% CI 3.39–4.34, PRR 3.51, IC 1.64), metabolic and nutritional disorders (ROR 1.65, 95% CI 1.02–2.69, PRR 1.65, IC 0.6), general cardiovascular disorders (ROR 6.77, 95% CI 5.23–8.76, PRR 6.6, IC 2.37), respiratory system disorders (ROR 3.57, 95% CI 2.99–4.25, PRR 3.43, IC 1.66), red blood cell disorders (ROR 4.14, 95% CI 2.86–6.00, PRR 3.53, IC 1.81), white cell and reticuloendothelial system (RES) disorder (ROR 4.02, 95% CI 3.52–4.61, PRR 3.73, IC 1.69), platelet, bleeding and clotting disorders (ROR 5.41, 95% CI 4.50–6.50, PRR 5.17, IC 2.06), body as a whole-general disorder (ROR 2.14, 95% CI 1.93–2.39, 1.97, IC 0.94) and urinary system disorders (ROR 5.56, 95% CI 4.76–6.50, PRR 5.22, IC 2.12) were more likely to be serious events (Table 3, Figure 2).

**Figure 3:**
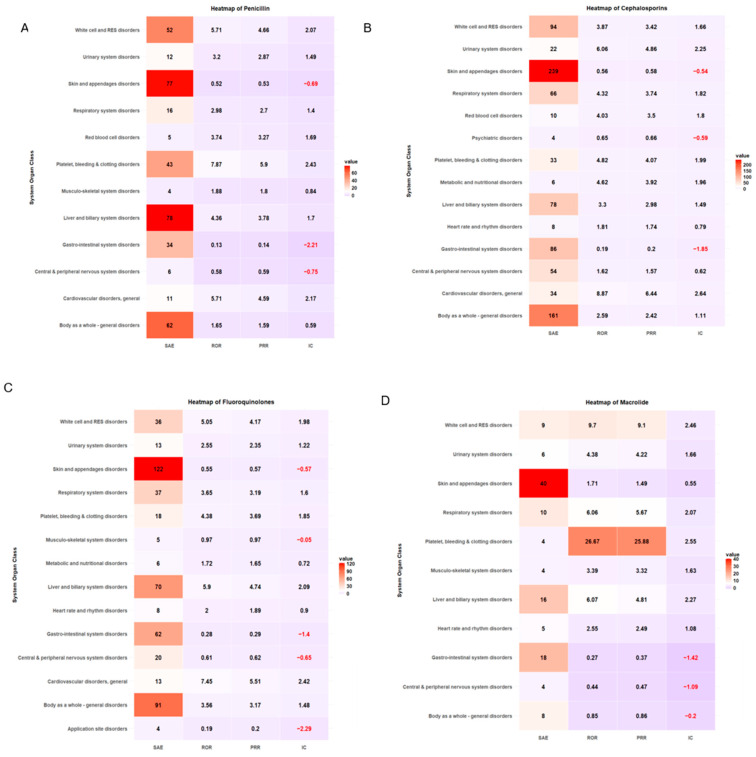

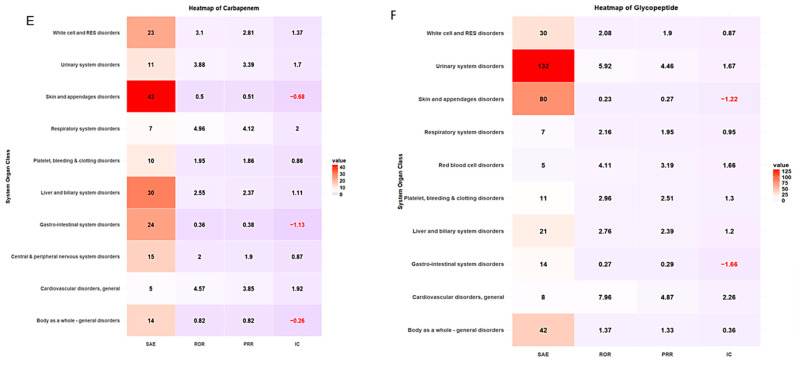
Heatmap of signal detection for antibacterial-associated ADEs. (**A**) penicillin, (**B**) cephalosporin, (**C**) fluoroquinolones, (**D**) macrolides, (**E**) carbapenems, and (**F**) glycopeptides.

ADE types classified by SOC and antibiotic class are summarized in Table 4. More than 20% of ADE cases were associated with skin and appendage disorders across antibiotic classes. The safety signal detection results of SOC-based ADEs, with their seriousness for each antibiotic class, are summarized and visualized in Table 5 and Figure 3, respectively. The strongest association between antibiotic classes and SAEs related to cardiovascular disorder was carbapenem (ROR 4.57, 95% CI 1.70–12.31, PRR 3.85, IC 1.92), cephalosporin (ROR 8.87, 95% CI 5.89–13.36, PRR 6.44, IC 2.64), fluoroquinolones (ROR 7.45, 95% CI 3.86–14.38, PRR 5.51, IC 2.42), and glycopeptide (ROR 7.96, 95% CI 3.12–20.30, PRR 4.87, IC 2.26) (Table 5, Figure 3). ADEs related to platelet, bleeding, and clotting disorders were more likely to be serious with penicillin disorders (ROR 7.87, 95% CI 5.44–11.39, PRR 5.90, IC 2.43) and macrolides (ROR 26.67, 95% CI 7.08–100.49, PRR 25.88, IC 2.55), while aminoglycosides had the strongest association with SAEs involving white cell and RES disorders (ROR 6.80, 95% CI 2.68–17.22, PRR 4.86, IC 2.21). A heatmap of other antibiotic classes is included in the supplementary (Supplementary Figures S1–S6).

A total of 34 ADEs were pertaining to antibiotic resistance mechanism disorders, with an estimated SAE prevalence of 8.22% (Table 6). Most ADEs related to antibiotic resistance mechanism disorders were reported with fluoroquinolones (n = 10, 29.4%), followed by carbapenems (n = 9, 26.47%).

### 3.3. Predictors Associated with Increased Risk of Antibiotic-Induced SAE Incidences

Univariate analysis identified patient age, number of concomitant medications and antibiotics, types of concomitant medications, and administration of specific antibacterial classes as predictors of increased risk of antibiotic-induced SAEs (Table 7). SAE risk increased with age (OR 1.05, 95% CI 1.03–1.07) and number of concomitant medications (OR 1.44, 95% CI 1.26–1.63). However, the risk of antibiotic-induced SAE is lower with multiple antibacterial therapies (OR 0.76, 95% CI 0.66–0.89). Concomitant administration of non-steroidal anti-inflammatory drugs (NSAIDs) (OR 1.38, 95% CI 1.26–1.63), proton pump inhibitors (PPIs) (OR 1.46, 95% CI 1.13–1.87), and anti-tuberculosis (TB) (OR 1.36, 95% CI 1.01–1.83) is more likely to increase SAEs, while concurrent administration of opioids (OR 0.25, 95% CI 0.16–0.38) is less likely to increase antibiotic-related SAE risks. Among numerous antibacterials, polymyxin B (OR 3.65, 95% CI 1.76–7.58) and sulfamethoxazole/trimethoprim (OR 3.14, 95% CI 2.37–4.16) revealed the highest association with increased SAE risks, followed by oxazolidinone (OR 2.76, 95% CI 1.68–4.52), glycopeptide (OR 1.98, 95% CI 1.76–2.23), lincomycin (OR 1.46, 95% CI 1.19–1.80), and aminoglycoside (OR 1.34, 95% CI 1.09–1.65). On the contrary, macrolide (OR 0.50, 95% CI 0.42–0.61) and tetracycline (OR 0.27, 95% CI 0.11–0.66) were less likely to develop serious antibiotic-induced ADEs.

### 3.4. Sensitivity Analysis on ADEs Related to Antibiotics Frequently Prescribed for MDR Gram-Negative Infection

**Figure 4:**
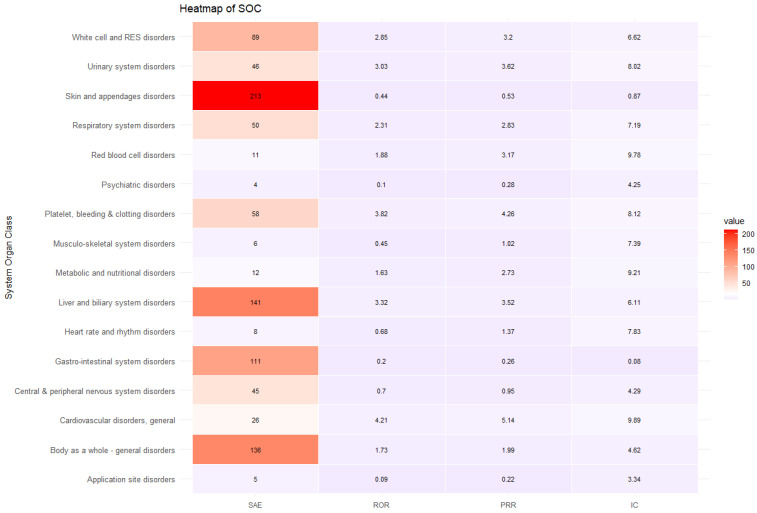
Heatmap of sensitivity analysis.

The sensitivity analysis included 18,677 ADE reports (35.57% of overall ADE cases included in the study), which included 975 SAE reports and 17,702 non-SAE reports. The sensitivity analysis revealed that ADEs were more likely to be serious in specific SOC classes involving general cardiovascular disease (ROR 6.62, 95% CI 4.21–10.40, PRR 5.14, IC 9.89), platelet, bleeding, and clotting disorders (ROR 5.14, 95% CI 3.82–6.93, PRR 4.26, IC 8.12), urinary system disorders (ROR 4.21, 95% CI 3.03–5.83, PRR 3.62, IC 8.02), liver and biliary system disorders (ROR 4.02, 95% CI 3.32–4.88, PRR 3.52, IC 6.11), white cell and RES disorders (ROR 3.61, 95% CI 2.85–4.57, PRR 3.20, IC 6.63), red blood cell disorders (ROR 3.60, 95% CI 1.88–6.89, PRR 3.17, IC 9.78), respiratory system disorder (ROR 3.14, 95% CI 2.31–4.26, PRR 2.83, IC 7.19), and metabolic and nutritional disorders (ROR 3.01, 95% CI 1.63–5.56, PRR 2.73, IC 9.22) (Table 8 and Figure 4).

The multivariate logistic regression revealed male sex (OR 1.18, 95% CI 1.03–1.35) and an increasing number of concomitant medications (OR 1.85, 95% CI 1.64–2.09) as predictors associated with increased SAE risks caused by antibiotics (Table 9). On the other hand, aging (OR 0.95, 95% CI 0.92–0.98), multiple antibiotic therapy (OR 0.63, 95% CI 0.53–0.75), and concomitant opioid use (OR 0.17, 95% CI 0.09–0.32) were associated with lower SAE risks. Among numerous antibacterial classes, aminoglycosides (OR 1.47, 95% CI 1.03–2.11), sulfamethoxazole/trimethoprim (OR 2.64, 95% CI 1.93–3.61), and polymyxin B (OR 3.21, 95% CI 1.50–6.76) were associated with higher SAE risks.

## 4. Discussion

This study provides a comprehensive pharmacovigilance analysis of antibacterial-induced ADEs utilizing a nationwide pharmacovigilance database, KIDS KAERS DB. Approximately 40% of antibiotic-related ADEs were reported in patients aged 60 years and older, with the majority of cases from patients aged 70 years and older. Cephalosporins, fluoroquinolones, and penicillin were among the most frequently implicated antibacterial classes, while glycopeptides, lincomycin, and polymyxin B were more likely to report SAEs. ADEs classified as liver and biliary system disorders, metabolic and nutritional disorders, cardiovascular disorders, respiratory system disorders, hematological disorders, and urinary system disorders were more likely to be serious. Importantly, predictors such as aging, an increasing number of concomitant medications, concomitant use of PPIs and NSAIDs, and specific antibacterial classes, particularly those with broad-spectrum activities, were substantially associated with higher SAE risk.

Consistent with high numbers of antibacterial prescriptions, most ADEs were reported with cephalosporins, fluoroquinolones, and penicillin [18]. Similar to previous studies [19], the majority of antibacterial-induced ADEs in this study were associated with skin and appendage disorders, responsible for at least 30% of all ADE cases of every antibiotic class. Nonetheless, the likelihood of the seriousness of these ADEs seemed significantly low. Additionally, fluoroquinolones were responsible for the majority of ADEs pertaining to antibacterial resistance mechanisms, followed by cephalosporins. Fluoroquinolones are broad-spectrum antibiotics with activity against antibiotic-resistant Gram-negative bacteria. However, the emergence of fluoroquinolone-resistant strains, especially in patients with urinary tract infections, resulted in regulation policy by many regulatory agencies [20]. Furthermore, recent pharmacovigilance studies suggest potential fluoroquinolone-induced psychiatric AEs [21], and similarly, this study demonstrated a relatively high prevalence of psychiatric ADEs with fluoroquinolones when compared to other antibacterial classes. Hence, this finding accentuates the importance of cautious prescribing and close monitoring of fluoroquinolones, particularly in vulnerable populations, such as those with higher resistance risks or pre-existing psychiatric conditions. Additionally, further research on the clinical significance as well as risk factor identification is warranted to provide evidence-based strategies to ensure the optimal and safe use of fluoroquinolones in clinical practice.

Consistent with previous studies, this study indicated that the risk of serious antibacterial-induced ADEs increases substantially with aging and the number of concomitant medications [22]. As aging is a major risk factor for many chronic diseases such as hypertension, diabetes mellitus, and hyperlipidemia, elderly patients not only have multiple comorbidities but also frequently administer several concomitant medications [23]. Among various drug classes, antibacterials are one of the most commonly prescribed classes in older adults when compared to younger adults [24]. Aging typically induces physiologic changes that alter pharmacokinetic and pharmacodynamic characteristics, making elderly patients more susceptible to SAEs [24,25]. Furthermore, polypharmacy, defined as the concurrent use of at least five medications daily, increases the risk of drug-induced SAEs [26]. Our previous pharmacovigilance studies also have shown a heightened risk of drug-induced SAEs with an increasing number of concomitant medications [14,15,27].

Interestingly, this study revealed decreased SAE risks with multiple antibacterial therapies. As combined antibiotic treatments can often increase the risk of developing ADEs, healthcare providers typically take extra caution and closely monitor patients when administering multiple antibiotics to mitigate potential risks, and this attentive oversight may have contributed to decreased SAE risk with an increasing number of concomitant antibacterial use [5]. Nonetheless, it is critical to consider that this reduced risk may not solely be attributed to cautious prescribing practice [5]. As patients receiving multiple antibiotics often present with more critical conditions, there may be some ADEs that go unrecognized or unreported due to the complexity of their illness [28]. Critically ill patients are usually susceptible to increased risk of drug interactions as they usually require a larger number of medications than non-critical patients, and concomitant administration of nephrotoxic or hepatic toxic medications can synergistically increase the ADE risks [28]. Moreover, these patients exhibit altered pharmacokinetics and pharmacodynamics, such as organ dysfunction secondary to impaired hepatic and renal function, which can lead to drug accumulation and accentuated risk of ADEs [29].

Additionally, the immune status of critically ill patients plays a critical factor. Clinical prognosis usually varies significantly depending on the type of infection, which accentuates the importance of accurate diagnosis and antibiotic susceptibility test results, as well as timely antibiotic treatments. For example, community-acquired pneumonia (CAP) tentatively has a more favorable prognosis than sepsis, which carries a substantially higher risk of morbidity and mortality due to potential multiple organ dysfunction and failure [30]. Sepsis is a common infection in critically ill patients, and those with severe immunosuppression may require broad-spectrum antibacterial [31]. Furthermore, complexity escalates when patients are diagnosed with MDR strains, as the patients with MDR infections are associated with negative clinical prognoses, manifested as extended hospital stays, increased healthcare costs, and limited treatment options [32]. MDR infections can also induce multiple complications that may obscure the differentiation between antibiotic-induced ADEs and complications arising from the infection [32]. For example, although hematologic ADEs are frequently reported with antibiotic use, causality determination may be challenging, particularly in sepsis patients, which usually induces numerous hematological changes [30]. These interactions and overlapping symptoms of serious illness may mask the identification of antibacterial-related ADEs, consequently leading to an underestimation of ADE risks in the population [28]. In this study, antibacterials with a spectrum of activity against resistant strains, including polymyxin B, oxazolidinone, glycopeptide, lincomycin, and aminoglycosides, were shown to increase the likelihood of serious antibacterial-induced ADEs. However, the causality of such clinical outcomes may remain unclear. Hence, while cautious monitoring is crucial, a comprehensive understanding of the overall condition of these patients is necessary to fully evaluate the implications of antibacterial therapy, particularly in critically ill patients.

This study included sensitivity analysis on the ADE cases of antibacterials commonly prescribed in MDR Gram-negative bacterial infections, as recommended in clinical guidelines [17], to specifically detect safety signals in critically ill patients, who are at higher risk for such infections. The sensitivity analysis, which included 35.57% of overall ADE cases, demonstrated similar trends in the association between SOC-based ADE cases and their seriousness [17]. However, the IC values, a potential indicator of the strength of association, were substantially higher than the overall analysis, and all SOC-based ADEs involving general cardiovascular disease, platelet, bleeding and clotting disorders, urinary system disorder, liver and biliary system disorders, white cell and RES disorders, red blood cell disorders, respiratory system disorder, and metabolic and nutritional disorders had IC > 3. While the higher IC values observed in certain SOC-based ADEs may reflect potential underlying disease, it is critical to note that the sensitivity analysis included all ADEs related to antibacterials commonly prescribed for MDR Gram-negative infection, regardless of the patients’ medical background, as the patient medical history was not fully available in the analysis. This limitation may have led to the inclusion of ADEs that may have occurred in patients without MDR Gram-negative infections or critical illness, potentially introducing bias. Hence, caution is required when interpreting these findings. Further studies, particularly cohort or controlled trials, are warranted to explore the role of comorbidities and patient medical history in the occurrence and seriousness of ADEs in critically ill patients. Given the complexity of this patient population, with multiple underlying medical conditions, polypharmacy, and altered pharmacokinetics, it is important to identify specific risk factors that contribute to the development of ADEs. Prospective cohort studies could provide more robust evidence by tracking ADE incidence in critically ill patients over time while controlling for factors like comorbidities, infection types, and concomitant medications, thereby providing evidence on the predictors associated with antibacterial SAEs. Additionally, controlled trials focusing on antibacterial therapy in critically ill patients could better isolate the direct effect of specific antibacterial therapy on ADE outcomes. Collectively, these approaches would provide a more comprehensive understanding of how comorbidities and medical history can contribute to the severity of ADEs, allowing for more personalized, evidence-based prescribing practices to improve patient safety in critically ill settings.

Types of concomitant medications are also essential predictors for SAEs associated with antibacterial use. In this study, the concomitant administration of NSAIDs and PPIs was associated with an increased likelihood of SAEs. Studies indicate that long-term use of PPIs may increase the risk of serious and rare ADEs, including cardiovascular disease, hepatic disease, renal disorders, and fractures [33]. Concomitant PPI use may induce several drug interactions by increasing gastric pH or inhibiting cytochrome P450 (CYP) 2C19 and 3A4, thereby impairing drug absorption and hepatic metabolism [34]. Previous studies revealed substantially higher mortality or ADE risks when PPIs were concomitantly used with antiplatelet or immune checkpoint inhibitors [35,36]. Furthermore, studies have suggested that concomitant treatment of antibiotics and PPIs is associated with poor outcomes in patients, manifested by a substantially elevated risk of *Clostridium difficile* infection (CDI) or increased toxicities [37,38]. On the other hand, NSAIDs are often used to manage fever associated with infections. NSAIDs can induce ADEs related to cardiovascular and renal toxicities, and concomitant NSAID use with antibacterials may elevate ADE risks, as the majority of antibacterials possess inherent cardiovascular and renal toxicity [39,40]. This finding emphasizes the importance of evaluating concomitant medication use to prevent risks associated with antibacterial use.

Concomitant opioid use with antibacterial was associated with reduced antibacterial-related SAE risks, which is both intriguing and counterintuitive. Opioids are usually prescribed to manage chronic pain, and our previous pharmacovigilance study on analgesics suggested a higher risk of SAEs from opioid use compared to NSAIDs [41], and reduced antibacterial-related SAE risks with concomitant opioid use is quite astonishing, which warrants further exploration. One plausible explanation is that patients on opioids often have severe medications, particularly cancer, where healthcare providers exercise additional caution in clinical settings [42]. Cancer patients may have compromised immune function, subsequently being at a higher risk of both infection and antibacterial-mediated ADEs [43]. As a result, healthcare providers may have extra caution on antibacterial administration and ADE monitoring, and enhanced surveillance in this population may contribute to the observed reduction in SAE risks [43,44]. Additionally, opioids are often prescribed after surgery or injury [45,46], situations in which antibiotics are frequently prescribed as prophylaxis or to treat infections. Healthcare professionals usually become particularly vigilant about potential ADEs, as post-operative patients often have multiple comorbidities, compromised immune systems, or impaired organ function, all of which could increase the risk of both infections and medication-related adverse events, and the heightened monitoring and careful medication selection in surgical or post-operative settings mitigate the risk of severe adverse events. Moreover, opioids may mask the presentation of certain adverse events, including gastrointestinal, respiratory, and cardiovascular symptoms, through their analgesic and sedative properties, thereby leading to underreporting or delayed recognition of ADEs [14,41]. While this masking effect might reduce the detection or reporting of specific adverse events, it also emphasizes the need for careful interpretation of pharmacovigilance data in patients on concomitant opioid and antibacterial therapy. Further research investigating the clinical impact of concomitant opioid use on the risk and seriousness of ADEs, particularly in vulnerable populations with complicated healthcare needs, is warranted.

As this study investigated ADEs primarily reported in Korea, caution is required when generalizing these findings to other regions or populations because variations in prescribing practices and resistance patterns across the regions could significantly impact the applicability of these findings in global settings. The prevalence of MDR organisms may vary widely by geographical region, influenced by local ASP policies, healthcare infrastructure, and regional epidemiological trends. Nevertheless, the findings of this study provide valuable insights and evidence on practical guidance on safe antibacterial use to prevent antibacterial-related ADEs, including SAEs. Clinicians should consider patient-specific factors such as age, comorbidities, and polypharmacy when prescribing antibiotics, as they were identified as potential predictors of increased SAE risks. Exclusive attention is needed when prescribing antibacterials with a broad spectrum of activities, as these agents were associated with a higher risk of SAEs. Tailoring antibiotic therapy to patients’ infections and individual risk factors is critical to improving patient outcomes. Additionally, healthcare professionals should be vigilant when managing medication lists, particularly NSAIDs and PPIs, which were demonstrated to increase the likelihood of SAEs when administered with antibacterials. Routine monitoring of renal and hepatic function is essential, particularly in critically ill patients or those on multiple medications, to prevent ADEs resulting from diseases or drug interactions, respectively. Furthermore, this study also suggests that, while multiple antibiotic therapy was associated with lower SAE risks due to potential vigilant monitoring, healthcare professionals must remain cautious, as critically ill patients may experience unrecognized or underreported ADEs due to the complexity of their conditions.

This is the first nationwide pharmacovigilance study on antibacterials using real-world data (RWD). The greatest benefit of RWD-utilized pharmacovigilance investigation is the generation of real-world evidence (RWE) on drug safety via large-scale post-marketing surveillance and rare ADR detection [14,15]. This study provides critical insights into antibacterial-induced ADEs, the seriousness of ADEs, and predictors of SAEs. However, this study possesses several limitations despite its essential finding on antibacterial-induced ADEs. First, as the KIDS KAERS DB is a spontaneous voluntary ADE reporting system, cautious interpretation is required secondary to the potential underreporting of ADEs and omissions of critical information such as comorbidities, indications, and concurrent medications. This may lead to incomplete or biased data, potentially influencing the validity and generalizability of the findings. Specifically, voluntary reporting systems are prone to underreporting, where less severe or less clinically obvious adverse events may not be documented, and selective reporting, where more serious ADEs are more likely to be reported. This can lead to an overrepresentation of SAEs, while mild or moderate ADEs may be underrepresented. Additionally, the quality and consistency of ADE report assessments, including the evaluation of seriousness, could be influenced by inter-reporter variability, as different healthcare providers may have varying criteria for assessing the seriousness of ADEs. Furthermore, as this study primarily relies on observational data, establishing causality remains challenging, particularly for critically ill patients. This is particularly relevant in infections like sepsis, where underlying medical conditions and infection-induced complications, such as hematological variations, may confound the attribution of ADEs to antibacterials. The limited availability of comprehensive patient-level data, such as comorbidity, indications, infection types, and concomitant medications, also hinders a more robust causality assessment, as the multifactorial nature of clinical contexts adds complexity to interpreting the relationship between antibacterials and ADEs. Furthermore, while several predictors of SAEs were identified, this analysis was not able to sufficiently explore interactions between these factors. Hence, further research is warranted to determine the evident causal relationship of predictors on increased SAE risks using patient-level data. Another important limitation is the generalizability of our findings. The data analyzed in this study are specific to the Korean healthcare system, which may limit the applicability of the results to other populations with different healthcare practices, bacterial resistance profiles, and pharmacovigilance systems. As the study relies on data from a single country’s pharmacovigilance system, caution should be exercised when attempting to extrapolate these findings to other regions, and future multi-national pharmacovigilance studies are warranted. Nonetheless, minimal bias from ADE cases was expected as the Korea Institute of Drug Safety and Risk Management (Ministry of Food and Drug Safety) performs in-depth investigations of reported ADEs by collecting scientific pharmacovigilance data from manufacturers, reviewing patients’ medical charts, and consulting with healthcare professionals appointed by the institution. Despite these limitations, this study contributes to the body of literature to guide safer antibacterial use and accentuates the importance and necessity for ongoing pharmacovigilance efforts and further research to enhance awareness of antibacterial-induced ADEs. Furthermore, this study provides tailored guidance on SAE prevention that can be endorsed as part of ASP, thereby enhancing patient safety and prognosis.

## 5. Conclusions

This study provided critical insights into antibacterial-associated ADEs and predictors of SAEs. Over 40% of antibacterial-related ADEs were reported in the elderly, aged 60 years and older, with a prevalence of serious antibacterial-induced ADEs at 5.60%. Most ADE cases were reported with cephalosporins, fluoroquinolones, and penicillin, primarily presented as skin and appendage disorders. Antibacterial classes with broad-spectrum activity against MDR bacteria, including glycopeptides, lincomycin, oxazolidinone, aminoglycosides, sulfamethoxazole/trimethoprim, and polymyxin B, were more frequently associated with SAEs. Key predictors of serious antibacterial-induced ADEs may include age, the number of concomitant medications, specific antibacterial classes, and types of concomitant medications such as PPIs and NSAIDs. However, ongoing ADE monitoring and future controlled or cohort studies remain crucial secondary to limited RWD-based pharmacovigilance investigations and the limitations of the study. Moreover, cautious monitoring, as well as tailored ASP, are critical to reducing antibacterial-associated ADEs. Routine monitoring of renal and hepatic function is essential, particularly in critically ill patients or those on multiple medications, to prevent ADEs resulting from diseases or drug interactions, respectively. Furthermore, this study also suggests that, while multiple antibiotic therapy was associated with lower SAE risks due to potential vigilant monitoring, healthcare professionals must remain cautious, as critically ill patients may experience unrecognized or underreported ADEs due to the complexity of their conditions. Hence, further studies are imperative to determine the causal relationship of antibacterial-induced ADEs in critically ill patients.

## Figures and Tables

**Table 1:** Baseline demographic characteristics of patients.

Characteristics	No of Cases (% Relative Frequency) or Median (IQR)
Age (years) ^a^	55 (34)
0~9	4254 (8.30%)
10~19	2305 (4.50%)
20~29	3437 (6.70%)
30~39	5011 (9.80%)
40~49	5806 (11.30%)
50~59	9672 (18.80%)
60~69	8771 (17.10%)
≥70	12,131 (23.60%)
Sex ^b^	
Men	24,983 (48.20%)
Women	26,839 (51.80%)
Causality	
Certain	1328 (2.50%)
Probable/Likely	17,399 (33.10%)
Possible	33,776 (64.30%)
Seriousness	
Serious adverse events	2917 (5.60%)
Nonserious adverse events	49,586 (94.40%)
Reporting individuals ^c^	
Doctors	12,738 (25.30%)
Pharmacists	5371 (10.70%)
Nurses and other healthcare professionals	26,939 (53.40%)
General Public	5369 (10.60%)
No. concomitantly used medications	
1	34,507 (65.72%)
2	11,605 (22.10%)
3	3911 (7.45%)
≥4	2480 (4.72%)
No. concomitantly used antibiotics	
1	36,561 (69.64%)
2	11,850 (22.57%)
3	2614 (4.98%)
≥4	1478 (2.82%)
Antibiotics types	
Penicillin	7623 (14.52%)
Cephalosporin	18,184 (34.63%)
Monobactam	45 (0.09%)
Carbapenem	3552 (6.77%)
Glycopeptide	3861 (7.35%)
Macrolide	4335 (8.26%)
Lincomycin	1396 (2.66%)
Tetracycline	367 (0.70%)
Oxazolidinone	131 (0.25%)
Aminoglycoside	1510 (2.88%)
Fluoroquinolone	9197 (17.52%)
Nitroimidazole	1767 (3.37%)
Sulfamethoxazole/Trimethoprim	378 (0.72%)
Mupirocin	104 (0.20%)
Polymyxin B	49 (0.09%)
Fusidic acid	4 (0.01%)

^a^ missing in 1116 (2.12%) cases. ^b^ missing in 681 (1.30.00%) cases. ^c^ missing in 2086 (3.97%) cases. Abbreviation: IQR: interquartile range.

**Table 2:** Association of antibiotic class with the seriousness of ADEs.

Antibiotic Classes	Non-SAE (N = 49,586)	SAE (N = 2917)	Total (N = 52,503)	*p*-Value	ROR (95%CI)
Penicillin	7217 (14.55%)	406 (13.92%)	7623 (14.52%)	0.343	0.950 (0.85–1.06)
Cephalosporin	17,282 (34.85%)	902 (30.92%)	18,184 (34.63%)	<0.001	0.84 (0.77–0.91)
Monobactam	40 (0.08%)	5 (0.17%)	45 (0.09%)	0.112	2.13 (0.84–5.4)
Carbapenem	3364 (6.78%)	188 (6.44%)	3552 (6.77%)	0.478	0.95 (0.81–1.10)
Glycopeptide	3502 (7.06%)	359 (12.31%)	3861 (7.35%)	<0.001	1.85 (1.65–2.07)
Macrolide	4205 (8.48%)	130 (4.46%)	4335 (8.26%)	<0.001	0.50 (0.42–0.60)
Lincomycin	1290 (2.60%)	106 (3.63%)	1396 (2.66%)	<0.001	1.41 (1.15–1.73)
Tetracycline	362 (0.73%)	5 (0.17%)	367 (0.70%)	0.001	0.23 (0.10–0.57)
Oxazolidinone	112 (0.23%)	19 (0.65%)	131 (0.25%)	<0.001	2.90 (1.78–4.72)
Aminoglycoside	1401 (2.83%)	109 (3.74%)	1510 (2.88%)	0.004	1.34 (1.10–1.63)
Fluoroquinolone	8681 (17.51%)	516 (17.69%)	9197 (17.52%)	0.801	1.01 (0.92–1.12)
Nitroimidazole	1671 (3.37%)	96 (3.29%)	1767 (3.37%)	0.818	0.98 (0.79–1.20)
Sulfamethoxazole/Trimethoprim	312 (0.63%)	66 (2.26%)	378 (0.72%)	<0.001	3.66 (2.80–4.78)
Mupirocin	104 (0.21%)	0 (0.00%)	104 (0.20%)	N/A	N/A
Polymyxin B	40 (0.08%)	9 (0.31%)	49 (0.09%)	<0.001	3.83 (1.86–7.91)
Fusidic acid	3 (0.01%)	1 (0.03%)	4 (0.01%)	N/A	N/A

Abbreviation: N/A: not applicable, ROR: reporting odds ratio, SAE: serious adverse events.

**Table 3:** System Organ Class (SOC)-based ADEs caused by antibiotics.

System Organ Class	Non-SAE (N = 49,586)	SAE (N = 2917)	Total (N = 52,503)	*p*-Value	ROR
Skin and appendage disorders	17,905 (36.11%)	702 (24.06%)	18,607 (35.44%)	0.001	0.56 (0.51–0.61)
Musculoskeletal system disorders	312 (0.63%)	18 (0.62%)	330 (0.63%)	0.936	0.98 (0.61–1.58)
Collagen disorders	9 (0.02%)	3 (0.10%)	12 (0.02%)	N/A	N/A
Central and peripheral nervous system disorders	2115 (4.27%)	120 (4.11%)	2235 (4.26%)	0.694	0.96 (0.80–1.16)
Autonomic nervous system disorders	0 (0.00%)	0 (0.00%)	0 (0.00%)	N/A	N/A
Vision disorders	182 (0.37%)	7(0.24%)	189 (0.36%)	0.269	0.65 (0.31–1.39)
Hearing and vestibular disorders	63 (0.13%)	4 (0.14%)	67 (0.13%)	0.882	1.08 (0.39–2.97)
Special sense other, disorders	133 (0.27%)	0 (0.00%)	133 (0.25%)	N/A	N/A
Psychiatric disorders	759 (1.53%)	8 (0.27%)	767 (1.46%)	<0.001	0.18 (0.09–0.36)
Gastrointestinal system disorders	17,127 (34.54%)	290 (9.94%)	17,417 (33.17%)	<0.001	0.21 (0.19–0.24)
Liver and biliary system disorders	1634 (3.30%)	337 (11.55%)	1971 (3.75%)	<0.001	3.83 (3.39–4.34)
Metabolic and nutritional disorders	185 (0.37%)	18 (0.62%)	203 (0.39%)	0.041	1.66 (1.02–2.69)
Endocrine disorders	3 (0.01%)	1 (0.03%)	4 (0.01%)	N/A	N/A
Cardiovascular disorders, general	211 (0.43%)	82 (2.81%)	293 (0.56%)	<0.001	6.77 (5.23–8.76)
Myo-, endo-, pericardial and valve disorders	13 (0.03%)	1 (0.03%)	14 (0.03%)	N/A	N/A
Heart rate and rhythm disorders	308 (0.62%)	25 (0.86%)	333 (0.63%)	0.120	1.38 (0.92–2.08)
Vascular (extracardiac) disorders	273 (0.55%)	5 (0.17%)	278 (0.53%)	0.01	0.31 (0.13–0.75)
Respiratory system disorders	789 (1.59%)	159 (5.45%)	948 (1.81%)	<0.001	3.57 (2.99–4.25)
Red blood cell disorders	145 (0.29%)	35 (1.20%)	180 (0.34%)	<0.001	4.14 (2.86–6.00)
White cell and RES disorders	1275 (2.57%)	280 (9.60%)	1555 (2.96%)	<0.001	4.02 (3.52–4.61)
Platelet, bleeding, and clotting disorders	506 (1.02%)	154 (5.28%)	660 (1.26%)	<0.001	5.40 (4.50–6.50)
Urinary system disorders	720 (1.45%)	221 (7.58%)	941 (1.79%)	<0.001	5.56 (4.76–6.50)
Reproductive disorders, male	3 (0.01%)	0 (0.00%)	3 (0.01%)	N/A	N/A
Reproductive disorders, female	11 (0.02%)	1 (0.03%)	12 (0.02%)	0.677	1.55 (0.20–11.98)
Neonatal and infancy disorders	5 (0.01%)	0 (0.00%)	5 (0.01%)	N/A	N/A
Neoplasms	9 (0.02%)	1 (0.03%)	10 (0.02%)	N/A	N/A
Body as a whole—general disorders	3767 (7.60%)	437 (14.98%)	4204 (8.01%)	<0.001	2.14 (1.93–2.39)
Application site disorders	1081 (2.18%)	5 (0.17%)	1086 (2.07%)	<0.001	0.08 (0.03–0.19)
Resistance mechanism disorders	31 (0.06%)	3 (0.10%)	34 (0.06%)	N/A	N/A
Secondary terms—events	10 (0.02%)	0 (0.00%)	10 (0.02%)	N/A	N/A
Poison specific terms	2 (0.00%)	0 (0.00%)	2 (0.00%)	N/A	N/A

N/A: not applicable.

**Table 4:** System Organ Class (SOC)-based ADEs classified by antibiotic classes.

	Penicillin		Cephalosporins		Monobactam		Carbapenem		Glycopeptide		Macrolide		Aminoglycoside		Fluoroquinolones	
	Non-SAE (n = 7217)	SAE (n = 406)	Non- SAE (n = 17,282)	SAE (n = 902)	Non-SAE (n = 40)	SAE (n = 5)	Non-SAE (n = 3364)	SAE (n = 188)	Non-SAE (n = 3502)	SAE (n = 359)	Non-SAE (n = 4205)	SAE (n = 130)	Non-SAE (n = 1401)	SAE (n = 109)	Non-SAE (n = 8681)	SAE (n = 516)
**Skin and appendage disorders**	2248 (31.15%)	77 (18.97%)	6754 (39.08%)	239 (26.50%)	22 (55.00%)	1 (20.00%)	1261 (37.49%)	43 (22.87%)	1926 (55.00%)	80 (22.28%)	869 (20.67%)	40 (30.77%)	428 (30.55%)	23 (21.10%)	3113 (35.86%)	122 (23.64%)
**Musculoskeletal system disorders**	38 (0.35%)	4 (0.99%)	102 (0.59%)	2 (0.22%)	0 (0.00%)	0 (0.00%)	9 (0.27%)	0 (0.00%)	10 (0.29%)	0 (0.00%)	39 (0.93%)	4 (3.08%)	10 (0.71%)	3 (2.75%)	87 (1.00%)	5 (0.97%)
**Collagen disorders**	0 (0.00%)	0 (0.00%)	3 (0.02%)	0 (0.00%)	0 (0.00%)	0 (0.00%)	2 (0.06%)	0 (0.00%)	2 (0.06%)	0 (0.00%)	0 (0.00%)	0 (0.00%)	0 (0.00%)	0 (0.00%)	0 (0.00%)	1 (0.19%)
**Central and peripheral nervous system disorders**	183 (2.54%)	6 (1.48%)	654 (3.78%)	54 (5.99%)	2 (5.00%)	0 (0.00%)	140 (4.16%)	15 (7.98%)	55 (1.57%)	3 (0.84%)	280 (6.66%)	4 (3.08%)	97 (6.92%)	6 (5.50%)	539 (6.21%)	20 (3.88%)
**Vision disorders**	7 (0.10%)	0 (0.00%)	33 (0.19%)	2 (0.22%)	0 (0.00%)	0 (0.00%)	0 (0.00%)	0 (0.00%)	2 (0.06%)	0 (0.00%)	7 (0.17%)	1 (0.77%)	24 (1.71%)	1 (0.92%)	84 (0.97%)	2 (0.39%)
**Hearing and vestibular disorders**	2 (0.03%)	0 (0.00%)	7 (0.04%)	0 (0.00%)	0 (0.00%)	0 (0.00%)	3 (0.09%)	0 (0.00%)	4 (0.11%)	0 (0.00%)	1 (0.02%)	0 (0.00%)	27 (1.93%)	3 (2.75%)	15 (0.17%)	1 (0.19%)
**Special sense other disorders**	11 (0.15%)	0 (0.00%)	8 (0.05%)	0 (0.00%)	0 (0.00%)	0 (0.00%)	3 (0.09%)	0 (0.00%)	0 (0.00%)	0 (0.00%)	83 (1.97%)	0 (0.00%)	0 (0.00%)	0 (0.00%)	13 (0.15%)	0 (0.00%)
**Psychiatric disorders**	46 (0.64%)	1 (0.25%)	117 (0.68%)	4 (0.44%)	0 (0.00%)	0 (0.00%)	18 (0.54%)	1 (0.53%)	19 (0.54%)	2 (0.56%)	282 (6.71%)	0 (0.00%)	13 (0.93%)	0 (0.00%)	206 (2.37%)	0 (0.00%)
**Gastrointestinal system disorders**	2926 (40.54%)	34 (8.37%)	6183 (35.78%)	86 (9.53%)	3 (7.50%)	0 (0.00%)	966 (28.72%)	24 (12.77%)	463 (13.22%)	14 (3.90%)	1584 (37.67%)	18 (13.85%)	482 (34.4%)	14 (12.84%)	2864 (32.99%)	62 (12.02%)
**Liver and biliary system disorders**	373 (5.17%)	78 (19.21%)	482 (2.79%)	78 (8.65%)	4 (10.00%)	1 (20.00%)	233 (6.93%)	30 (15.96%)	77 (2.20%)	21 (5.85%)	95 (2.26%)	16 (12.31%)	40 (2.86%)	16 (14.68%)	225 (2.59%)	70 (13.57%)
**Metabolic and nutritional disorders**	21 (0.29%)	0 (0.00%)	25 (0.14%)	6 (0.67%)	0 (0.00%)	0 (0.00%)	12 (036%)	1 (0.53%)	24 (0.69%)	3 (0.84%)	13 (0.31%)	1 (0.77%)	10 (0.71%)	0 (0.00%)	59 (0.68%)	6 (1.16%)
**Endocrine disorders**	1 (0.00%)	0 (0.00%)	0 (0.00%)	0 (0.00%)	0 (0.00%)	0 (0.00%)	0 (0.00%)	0 (0.00%)	1 (0.03%)	0 (0.00%)	0 (0.00%)	0 (0.00%)	1 (0.07%)	0 (0.00%)	0 (0.00%)	1 (0.19%)
**Cardiovascular disorders, general**	35 (0.48%)	11 (2.71%)	76 (0.44%)	34 (3.77%)	1 (2.50%)	0 (0.00%)	20 (0.59%)	5 (2.66%)	10 (0.29%)	8 (2.23%)	13 (0.31%)	2 (1.54%)	15 (1.07%)	4 (3.67%)	30 (0.35%)	13 (2.52%)
**Myo-, endo-, pericardial, and valve disorders**	2 (0.03%)	0 (0.00%)	5 (0.03%)	1 (0.11%)	0 (0.00%)	0 (0.00%)	0 (0.00%)	0 (0.00%)	0 (0.00%)	0 (0.00%)	1 (0.02%)	0 (0.00%)	0 (0.00%)	0 (0.00%)	2 (0.02%)	0 (0.00%)
**Heart rate and rhythm disorders**	41 (0.57%)	3 (0.74%)	85 (0.49%)	8 (0.89%)	0 (0.00%)	0 (0.00%)	8 (0.24%)	0 (0.00%)	20 (0.57%)	0 (0.00%)	65 (1.55%)	5 (3.85%)	6 (0.43%)	0 (0.00%)	68 (0.78%)	8 (1.55%)
**Vascular (extracardiac) disorders**	28 (0.39%)	1 (0.25%)	116 (0.67%)	0 (0.00%)	0 (0.00%)	0 (0.00%)	9 (0.27%)	0 (0.00%)	16 (0.46%)	1 (0.28%)	41 (0.98%)	0 (0.00%)	5 (0.36%)	0 (0.00%)	46 (0.53%)	3 (0.58%)
**Respiratory system disorders**	98 (1.36%)	16 (3.94%)	310 (1.79%)	66 (7.32%)	0 (0.00%)	0 (0.00%)	26 (0.77%)	7 (3.72%)	32 (0.91%)	7 (1.95%)	57 (1.36%)	10 (7.69%)	42 (3.00%)	3 (2.75%)	180 (2.07%)	37 (7.17%)
**Red blood cell disorders**	24 (0.33%)	5 (1.23%)	48 (0.28%)	10 (1.11%)	0 (0.00%)	0 (0.00%)	30 (0.89%)	3 (1.60%)	12 (0.34%)	5 (1.39%)	1 (0.02%)	2 (1.54%)	1 (0.07%)	1 (0.92%)	15 (0.17%)	2 (0.39%)
**White cell and RES disorders**	181 (2.51%)	52 (12.81%)	504 (2.92%)	94 (10.42%)	1 (2.50%)	0 (0.00%)	145 (4.31%)	23 (12.23%)	147 (4.20%)	30 (8.36%)	32 (0.76%)	9 (6.92%)	14 (1.00%)	7 (6.42%)	127 (1.46%)	36 (6.98%)
**Platelet, bleeding, and clotting disorders**	107 (1.48%)	43 (10.59%)	135 (0.78%)	33 (3.66%)	1 (2.50%)	1 (20.00%)	94 (2.79%)	10 (5.32%)	37 (1.06%)	11 (3.06%)	5 (0.12%)	4 (3.08%)	3 (0.21%)	2 (1.83%)	71 (0.82%)	18 (3.49%)
**Urinary system disorders**	68 (0.94%)	12 (2.96%)	71 (0.41%)	22 (2.44%)	1 (2.50%)	1 (20.00%)	53 (1.58%)	11 (5.85%)	313 (8.94%)	132 (36.77%)	46 (1.09%)	6 (4.62%)	42 (3.00%)	13 (11.93%)	87 (1.00%)	13 (2.52%)
**Reproductive disorders, male**	0 (0.00%)	0 (0.00%)	1 (0.01%)	0 (0.00%)	0 (0.00%)	0 (0.00%)	0 (0.00%)	0 (0.00%)	0 (0.00%)	0 (0.00%)	1 (0.02%)	0 (0.00%)	0 (0.00%)	0 (0.00%)	0 (0.00%)	0 (0.00%)
**Reproductive disorders, female**	1 (0.01%)	0 (0.00%)	2 (0.01%)	1 (0.11%)	0 (0.00%)	0 (0.00%)	2 (0.06%)	0 (0.00%)	0 (0.00%)	0 (0.00%)	0 (0.00%)	0 (0.00%)	0 (0.00%)	0 (0.00%)	6 (0.07%)	0 (0.00%)
**Neonatal and infancy disorder**	1 (0.01%)	0 (0.00%)	2 (0.01%)	0 (0.00%)	0 (0.00%)	0 (0.00%)	0 (0.00%)	0 (0.00%)	1 (0.03%)	0 (0.00%)	0 (0.00%)	0 (0.00%)	0 (0.00%)	0 (0.00%)	1 (0.01%)	0 (0.00%)
**Neoplasms**	1 (0.01%)	1 (0.25%)	4 (0.02%)	0 (0.00%)	0 (0.00%)	0 (0.00%)	1 (0.03%)	0 (0.00%)	1 (0.03%)	0 (0.00%)	0 (0.00%)	0 (0.00%)	0 (0.00%)	0 (0.00%)	0 (0.00%)	0 (0.00%)
**Body as a whole—general disorders**	712 (9.87%)	62 (15.27%)	1339 (7.75%)	161 (17.85%)	5 (12.50%)	1 (20.00%)	302 (8.98%)	14 (7.45%)	309 (8.82%)	42 (11.70%)	300 (7.13%)	8 (6.15%)	107 (7.64%)	13 (11.93%)	492 (5.67%)	91 (17.64%)
**Application site disorders**	59 (0.82%)	0 (0.00%)	205 (1.19%)	0 (0.00%)	0 (0.00%)	0 (0.00%)	18 (0.54%)	1 (0.53%)	19 (0.54%)	0 (0.00%)	387 (9.20%)	0 (0.00%)	33 (2.36%)	0 (0.00%)	342 (3.94%)	4 (0.78%)
**Resistance mechanism disorders**	2 (0.03%)	0 (0.00%)	7 (0.04%)	1 (0.11%)	0 (0.00%)	0 (0.00%)	9 (0.27%)	0 (0.00%)	1 (0.03%)	0 (0.00%)	0 (0.00%)	0 (0.00%)	0 (0.00%)	0 (0.00%)	9 (0.10%)	1 (0.19%)
**Secondary terms—events**	1 (0.01%)	0 (0.00%)	4 (0.02%)	0 (0.00%)	0 (0.00%)	0 (0.00%)	0 (0.00%)	0 (0.00%)	1 (0.03%)	0 (0.00%)	3 (0.07%)	0 (0.00%)	1 (0.07%)	0 (0.00%)	0 (0.00%)	0 (0.00%)
**Poison** **-** **specific term**	0 (0.00%)	0 (0.00%)	0 (0.00%)	0 (0.00%)	0 (0.00%)	0 (0.00%)	0 (0.00%)	0 (0.00%)	0 (0.00%)	0 (0.00%)	0 (0.00%)	0 (0.00%)	0 (0.00%)	0 (0.00%)	0 (0.00%)	0 (0.00%)
	**Tetracyclines**		**Lincomycin**		**Nitroimidazole**		**Sulfamethoxazole/Trimethoprim**		**Mupirocin**		**Polym** **y** **xin B**		**Fusidine**		**Oxazolidinones**	
	**Non-SAE** **(n = 362)**	**SAE** **(n = 5)**	**Non-SAE** **(n = 1290)**	**SAE** **(n = 106)**	**Non-SAE** **(n = 1671)**	**SAE** **(n = 96)**	**Non-SAE** **(n = 312)**	**SAE** **(n = 66)**	**Non-SAE** **(n = 104)**	**SAE** **(n = 0)**	**Non-SAE** **(n = 40)**	**SAE** **n = 9)**	**Non-SAE** **(n = 3)**	**SAE** **(n = 1)**	**Non-SAE** **(n = 112)**	**SAE** **(n = 9)**
**Skin and appendage disorders**	107 (29.56%)	4 (80.00%)	541 (41.94%)	35 (33.02%)	497 (29.74%)	18 (18.75%)	90 (28.85%)	14 (21.21%)	16 (15.38%)	0 (0.00%)	7 (17.50%)	5 (55.56%)	2 (66.67%)	0 (0.00%)	24 (21.43%)	1 (5.26%)
**Musculoskeletal system disorders**	1 (0.28%)	0 (0.00%)	7 (0.54%)	0 (0.00%)	6 (0.36%)	0 (0.00%)	0 (0.00%)	0 (0.00%)	0 (0.00%)	0 (0.00%)	1 (2.50%)	0 (0.00%)	0 (0.00%)	0 (0.00%)	2 (1.79%)	0 (0.00%)
**Collagen disorders**	0 (0.00%)	0 (0.00%)	1 (0.08%)	0 (0.00%)	1 (0.06%)	0 (0.00%)	0 (0.00%)	2 (3.03%)	0 (0.00%)	0 (0.00%)	0 (0.00%)	0 (0.00%)	0 (0.00%)	0 (0.00%)	0 (0.00%)	0 (0.00%)
**Central and peripheral nervous system disorders**	8 (2.21%)	0 (0.00%)	62 (4.81%)	7 (6.6%)	79 (4.73%)	5 (5.21%)	6 (1.92%)	0 (0.00%)	2 (1.92%)	0 (0.00%)	2 (5.00%)	0 (0.00%)	0 (0.00%)	0 (0.00%)	6 (5.36%)	0 (0.00%)
**Vision disorders**	18 (4.97%)	0 (0.00%)	1 (0.08%)	0 (0.00%)	2 (0.12%)	0 (0.00%)	0 (0.00%)	0 (0.00%)	1 (0.96%)	0 (0.00%)	3 (7.50%)	1 (11.11%)	0 (0.00%)	0 (0.00%)	0 (0.00%)	0 (0.00%)
**Hearing and vestibular disorders**	2 (0.55%)	0 (0.00%)	1 (0.08%)	0 (0.00%)	1 (0.06%)	0 (0.00%)	0 (0.00%)	0 (0.00%)	0 (0.00%)	0 (0.00%)	0 (0.00%)	0 (0.00%)	0 (0.00%)	0 (0.00%)	0 (0.00%)	0 (0.00%)
**Special sense other disorders**	0 (0.00%)	0 (0.00%)	6 (0.47%)	0 (0.00%)	7 (0.42%)	0 (0.00%)	2 (0.64%)	0 (0.00%)	0 (0.00%)	0 (0.00%)	0 (0.00%)	0 (0.00%)	0 (0.00%)	0 (0.00%)	0 (0.00%)	0 (0.00%)
**Psychiatric disorders**	2 (0.55%)	0 (0.00%)	15 (1.16%)	0 (0.00%)	18 (1.08%)	0 (0.00%)	10 (3.21%)	0 (0.00%)	12 (11.54%)	0 (0.00%)	1 (2.50%)	0 (0.00%)	0 (0.00%)	0 (0.00%)	0 (0.00%)	0 (0.00%)
**Gastrointestinal system disorders**	176 (48.62%)	0 (0.00%)	432 (33.49%)	15 (14.15%)	864 (51.71%)	18 (18.75%)	86 (27.56%)	0 (0.00%)	43 (41.35%)	0 (0.00%)	12 (30.00%)	3 (33.33%)	0 (0.00%)	0 (0.00%)	43 (38.39%)	2 (10.53%)
**Liver and biliary system disorders**	4 (1.1%)	1 (20.00%)	58 (4.50%)	14 (13.21%)	13 (0.78%)	6 (6.25%)	24 (7.69%)	4 (6.06%)	0 (0.00%)	0 (0.00%)	0 (0.00%)	0 (0.00%)	1 (33.33%)	0 (0.00%)	5 (4.46%)	2 (10.53%)
**Metabolic and nutritional disorders**	1 (0.28%)	0 (0.00%)	6 (0.47%)	0 (0.00%)	10 (0.60%)	0 (0.00%)	2 (0.64%)	0 (0.00%)	1 (0.96%)	0 (0.00%)	1 (2.50%)	0 (0.00%)	0 (0.00%)	0 (0.00%)	0 (0.00%)	1 (5.26%)
**Endocrine disorders**	0 (0.00%)	0 (0.00%)	0(0.00%)	0 (0.00%)	0 (0.00%)	0 (0.00%)	0 (0.00%)	0 (0.00%)	0 (0.00%)	0 (0.00%)	0 (0.00%)	0 (0.00%)	0 (0.00%)	0 (0.00%)	0 (0.00%)	0 (0.00%)
**Cardiovascular disorders, general**	0 (0.00%)	0 (0.00%)	4 (0.31%)	3 (2.83%)	3 (0.18%)	0 (0.00%)	4 (1.28%)	2 (3.03%)	0 (0.00%)	0 (0.00%)	0 (0.00%)	0 (0.00%)	0 (0.00%)	0 (0.00%)	0 (0.00%)	0 (0.00%)
**Myo-, endo-, pericardial, and valve disorders**	0 (0.00%)	0 (0.00%)	2 (0.16%)	0 (0.00%)	1 (0.06%)	0 (0.00%)	0 (0.00%)	0 (0.00%)	0 (0.00%)	0 (0.00%)	0 (0.00%)	0 (0.00%)	0 (0.00%)	0 (0.00%)	0 (0.00%)	0 (0.00%)
**Heart rate and rhythm disorders**	0 (0.00%)	0 (0.00%)	6 (0.47%)	1 (0.94%)	5 (0.30%)	0 (0.00%)	4 (1.28%)	0 (0.00%)	0 (0.00%)	0 (0.00%)	0 (0.00%)	0 (0.00%)	0 (0.00%)	0 (0.00%)	0 (0.00%)	0 (0.00%)
**Vascular (extracardiac) disorders**	0 (0.00%)	0 (0.00%)	8 (0.62%)	0 (0.00%)	3 (0.18%)	0 (0.00%)	0 (0.00%)	0 (0.00%)	0 (0.00%)	0 (0.00%)	1 (2.50%)	0 (0.00%)	0 (0.00%)	0 (0.00%)	0 (0.00%)	0 (0.00%)
**Respiratory system disorders**	1 (0.28%)	0 (0.00%)	11 (0.85%)	10 (9.43%)	20 (1.20%)	2 (2.08%)	2 (0.64%)	0 (0.00%)	6 (5.77%)	0 (0.00%)	3 (7.50%)	0 (0.00%)	0 (0.00%)	0 (0.00%)	1 (0.89%)	1 (5.26%)
**Red blood cell disorders**	0 (0.00%)	0 (0.00%)	6 (0.47%)	0 (0.00%)	4 (0.24%)	1 (1.04%)	2 (0.64%)	4 (6.06%)	0 (0.00%)	0 (0.00%)	0 (0.00%)	0 (0.00%)	0 (0.00%)	0 (0.00%)	2 (1.79%)	2 (10.53%)
**White cell and RES disorders**	25 (6.91%)	0 (0.00%)	23 (1.78%)	5 (4.72%)	39 (2.33%)	12 (12.50%)	14 (4.49%)	12 (18.18%)	16 (15.38%)	0 (0.00%)	0 (0.00%)	0 (0.00%)	0 (0.00%)	0 (0.00%)	7 (6.25%)	10 (52.63%)
**Platelet, bleeding, and clotting disorders**	1 (0.28%)	0 (0.00%)	6 (0.47%)	3 (2.83%)	18 (1.08%)	3 (3.13%)	12 (3.85%)	16 (24.24%)	0 (0.00%)	0 (0.00%)	0 (0.00%)	0 (0.00%)	0 (0.00%)	0 (0.00%)	16 (14.29%)	0 (0.00%)
**Urinary system disorders**	2 (0.55%)	0 (0.00%)	3 (0.23%)	3 (2.83%)	9 (0.54%)	2 (2.08%)	24 (7.69%)	6 (9.09%)	0 (0.00%)	0 (0.00%)	0 (0.00%)	0 (0.00%)	0 (0.00%)	0 (0.00%)	1 (0.89%)	0 (0.00%)
**Reproductive disorders, male**	0 (0.00%)	0 (0.00%)	0 (0.00%)	0 (0.00%)	1 (0.06%)	0 (0.00%)	0 (0.00%)	0 (0.00%)	0 (0.00%)	0 (0.00%)	0 (0.00%)	0 (0.00%)	0 (0.00%)	0 (0.00%)	0 (0.00%)	0 (0.00%)
**Reproductive disorders, female**	0 (0.00%)	0 (0.00%)	0 (0.00%)	0 (0.00%)	0 (0.00%)	0 (0.00%)	0 (0.00%)	0 (0.00%)	0 (0.00%)	0 (0.00%)	0 (0.00%)	0 (0.00%)	0 (0.00%)	0 (0.00%)	0 (0.00%)	0 (0.00%)
**Neonatal and infancy disorders**	0 (0.00%)	0 (0.00%)	0 (0.00%)	0 (0.00%)	0 (0.00%)	0 (0.00%)	0 (0.00%)	0 (0.00%)	0 (0.00%)	0 (0.00%)	0 (0.00%)	0 (0.00%)	0 (0.00%)	0 (0.00%)	0 (0.00%)	0 (0.00%)
**Neoplasms**	0 (0.00%)	0 (0.00%)	1 (0.08%)	0 (0.00%)	1 (0.06%)	0 (0.00%)	0 (0.00%)	0 (0.00%)	0 (0.00%)	0 (0.00%)	0 (0.00%)	0 (0.00%)	0 (0.00%)	0 (0.00%)	0 (0.00%)	0 (0.00%)
**Body as a whole—general disorders**	13 (3.59%)	0 (0.00%)	82 (6.36%)	10 (9.43%)	62 (3.71%)	28 (29.17%)	28 (8.97%)	6 (9.09%)	7 (6.73%)	0 (0.00%)	6 (15.50%)	0 (0.00%)	0 (0.00%)	1 (100%)	3 (2.68%)	0 (0.00%)
**Application site disorders**	0 (0.00%)	0 (0.00%)	8 (0.62%)	0 (0.00%)	6 (0.36%)	0 (0.00%)	0 (0.00%)	0 (0.00%)	0 (0.00%)	0 (0.00%)	3 (7.50%)	0 (0.00%)	0 (0.00%)	0 (0.00%)	1 (0.89%)	0 (0.00%)
**Resistance mechanism disorders**	1 (0.28%)	0 (0.00%)	0 (0.00%)	0 (0.00%)	1 (0.06%)	1 (1.04%)	0 (0.00%)	0 (0.00%)	0 (0.00%)	0 (0.00%)	0 (0.00%)	0 (0.00%)	0 (0.00%)	0 (0.00%)	1 (0.89%)	0 (0.00%)
**Secondary terms—events**	0 (0.00%)	0 (0.00%)	0 (0.00%)	0 (0.00%)	0 (0.00%)	0 (0.00%)	0 (0.00%)	0 (0.00%)	0 (0.00%)	0 (0.00%)	0 (0.00%)	0 (0.00%)	0 (0.00%)	0 (0.00%)	0 (0.00%)	0 (0.00%)
**Poison** **-** **specific term**	0 (0.00%)	0 (0.00%)	0 (0.00%)	0 (0.00%)	0 (0.00%)	0 (0.00%)	2 (0.64%)	0 (0.00%)	0 (0.00%)	0 (0.00%)	0 (0.00%)	0 (0.00%)	0 (0.00%)	0 (0.00%)	0 (0.00%)	0 (0.00%)

**Table 5:** Association of SOC-based ADEs with its seriousness for each antibiotic class.

System Organ Class	Penicillin	Cephalosporin	Carbapenem	Glycopeptide	Macrolides	Aminoglycosides	Fluoroquinolones	Tetracyclines	Lincomycin	Nitroimidazole	Sulfamethoxazole/Trimethoprim	Polymyxin B	Oxazolidinones
Skin and appendage disorders	**0.52 (0.40–0.67)**	**0.56 (0.48–0.65)**	**0.50 (0.35–0.70)**	**0.24 (0.18–0.30)**	**1.71 (1.17–2.50)**	**0.61 (0.38–0.98)**	**0.55 (0.45–0.68)**	**9.53 (1.05–86.28)**	0.68 (0.45–1.04)	**0.55 (0.32–0.92)**	0.66 (0.35–1.26)	**5.89 (1.25–27.69)**	N/A
Musculoskeletal system disorders	1.88 (0.67–5.29)	N/A	N/A	N/A	**3.39 (1.19–9.63)**	N/A	0.97 (0.39–2.39)	N/A	N/A	N/A	N/A	N/A	N/A
Central and peripheral nervous system disorders	0.58 (0.25–1.31)	**1.62 (1.22–2.16)**	**2.00 (1.15–3.48)**	N/A	0.45 (0.16–1.21)	0.78 (0.34–1.83)	**0.61 (0.39–0.96)**	N/A	1.40 (0.62–3.14)	1.11 (0.44–2.80)	N/A	N/A	N/A
Psychiatric disorders	N/A	0.65 (0.24–1.77)	N/A	N/A	N/A	N/A	N/A	N/A	N/A	N/A	N/A	N/A	N/A
Gastrointestinal system disorders	**0.13 (0.09–0.19)**	**0.19 (0.15–0.24)**	**0.36 (0.24–0.56)**	**0.26 (0.16–0.46)**	**0.27 (0.16–0.44)**	**0.28 (0.16–0.50)**	**0.28 (0.21–0.36)**	N/A	**0.33 (0.19–0.57)**	**0.22 (0.13–0.36)**	N/A	N/A	N/A
Liver and biliary system disorders	**4.36 (3.34–5.70)**	**3.30 (2.57–4.23)**	**2.55 (1.70–3.85)**	**2.76 (1.68–4.54)**	**6.07 (3.46–10.65)**	**5.85 (3.16–10.85)**	**5.90 (4.44–7.84)**	N/A	**3.23 (1.74–6.01)**	**8.50 (3.16–22.89)**	0.77 (0.26–2.31)	N/A	N/A
Metabolic and nutritional disorders	N/A	**4.62 (1.89–11.30)**	N/A	N/A	N/A	N/A	1.72 (0.74–4.00)	N/A	N/A	N/A	N/A	N/A	N/A
Cardiovascular disorders, general	**5.71 (2.88–11.34)**	**8.87 (5.89–13.36)**	**4.57 (1.70–12.31)**	**7.96 (3.12–20.30)**	N/A	**3.52 (1.15–10.80)**	**7.45 (3.86–14.38)**	N/A	N/A	N/A	N/A	N/A	N/A
Heart rate and rhythm disorders	N/A	**1.81 (0.87–3.75)**	N/A	N/A	**2.55 (1.01 -6.44)**	N/A	2.00 (0.97–4.17)	N/A	N/A	N/A	N/A	N/A	N/A
Respiratory system disorders	**2.98 (1.74–5.11)**	**4.32 (3.28–5.69)**	**4.97 (2.13–11.59)**	2.16 (0.95–4.92)	**6.06 (3.02–12.16)**	N/A	**3.65 (2.53–5.26)**	N/A	**12.11 (5.02–29.23)**	N/A	N/A	N/A	N/A
Red blood cell disorders	**3.74 (1.42–9.85)**	**4.03 (2.03–7.98)**	N/A	**4.11 (1.44–11.73)**	N/A	N/A	N/A	N/A	N/A	N/A	**10.00 (1.79–55.80)**	N/A	N/A
White cell and RES disorders	**5.71 (4.12–7.91)**	**3.87 (3.07–4.88)**	**3.10 (1.94–4.94)**	**2.08 (1.38–3.13)**	**9.70 (4.53–20.77)**	**6.80 (2.68–17.22)**	**5.05 (3.45–7.40)**	N/A	**2.73 (1.02–7.33)**	**5.98 (3.02–11.84)**	**4.73 (2.08–10.78)**	N/A	**16.67 (5.11–54.33)**
Platelet, bleeding, and clotting disorders	**7.87 (5.44–11.39)**	**4.82 (3.28–7.10)**	**1.95 (1.00–3.82)**	**2.96 (1.50–5.86)**	**26.67 (7.08–100.49)**	N/A	**4.38 (2.59–7.41)**	N/A	N/A	N/A	**8.00 (3.57–17.91)**	N/A	N/A
Urinary system disorders	**3.20 (1.72–5.96)**	**6.06 (3.74–9.82)**	**3.88 (1.99–7.56)**	**5.93 (4.64–7.56)**	**4.38 (1.83–10.44)**	**4.38 (2.28–8.44)**	**2.55 (1.42–4.60)**	N/A	N/A	N/A	1.20 (0.47–3.06)	N/A	N/A
Body as a whole—general disorders	**1.65 (1.24–2.18)**	**2.59 (2.16–3.10)**	0.82 (0.47–1.42)	1.37 (0.97–1.93)	0.85 (0.41–1.76)	1.64 (0.89–3.02)	**3.56 (2.79–4.55)**	N/A	1.54 (0.77–3.06)	**10.69 (6.43–17.76)**	1.01 (0.40–2.56)	N/A	N/A
Application site disorders	N/A	N/A	N/A	N/A	N/A	N/A	**0.19 (0.07–0.51)**	N/A	N/A	N/A	N/A	N/A	N/A

Bold texts indicate statistical significance. N/A indicates not applicable.

**Table 6:** ADE cases pertaining to antibiotic resistance mechanism disorders (n = 34).

Sex ^a^	
Men	18 (52.94%)
Women	14 (41.18%)
Seriousness	
SAEs	3 (8.22%)
Non-SAEs	31 (91.18%)
Antibiotic Types	
Penicillin	
Ampicillin	1 (2.94%)
Piperacillin/Tazobactam	1 (2.94%)
Carbapenem	
Imipenem/Cilastatin	8 (23.52%)
Meropenem	1 (2.94%)
Cephalosporins	
Cefadroxil	1 (2.94%)
Cefdinir	1 (2.94%)
Cefditoren	1 (2.94%)
Cefepime	1 (2.94%)
Cefotetan	1 (2.94%)
Cefotaxime	1 (2.94%)
Ceftazidime	1 (2.94%)
Ceftriaxone	1 (2.94%)
Fluoroquinolones	
Ciprofloxacin	5 (14.70%)
Levofloxacin	4 (11.76%)
Ofloxacin	1 (2.94%)
Tetracyclines	
Doxycycline	1 (2.94%)
Oxazolidinones	
Linezolid	1 (2.94%)
Nitroimidazole	
Metronidazole	2 (5.88%)
Glycopeptides	
Vancomycin	1 (2.94%)

^a^ missing in 2 (5.88%) cases.

**Table 7:** Univariate and multivariate analyses for the association between predictors and serious adverse events (SAEs).

Predictors	Univariate Logistic Regression (Number of Cases (%))			Multivariate Logistic Regression Analysis	
	SAEs (n = 2917)	Non-SAEs (n = 49,586)	*p*-Value	OR (95% CI)	*p*-Value
Sex					
Male	1396 (2.7%)	23,587 (45.5%)	N/A	N/A	
Female	1472 (2.8%)	25,367 (49.0%)			
Age					
0–9	141 (0.3%)	4113 (8.0%)	<0.001	1.05 (1.03–1.07)	<0.001
10–19	115 (0.2%)	2190 (4.3%)			
20–29	206 (0.4%)	3231 (6.3%)			
30–39	309 (0.6%)	4702 (9.2%)			
40–49	345 (0.7%)	5461 (10.6%)			
50–59	454 (0.9%)	9218 (17.9%)			
60–69	503 (1.0%)	8268 (16.1%)			
≥70	771 (1.5%)	11,360 (22.1%)			
Causality					
Certain	73 (0.1%)	1255 (2.4%)	N/A	N/A	
Probable/likely	944 (1.8%)	16,455 (31.3%)			
Possible	1900 (3.6%)	31,876 (60.7%)			
Number of concurrently used medications					
1	1664 (3.2%)	32,843 (62.6%)	<0.001	1.44 (1.26–1.63)	<0.001
2	683 (1.3%)	10,922 (20.8%)			
3	401 (0.8%)	3510 (6.7%)			
≥4	169 (0.3%)	2311(4.4%)			
Number of used antibacterial					
1	1853 (3.5%)	34,708 (66.1%)	<0.001	0.76 (0.66–0.89)	<0.001
2	771 (1.5%)	11,079 (21.1%)			
≥3	293 (0.6%)	3799 (7.2%)			
Types of concomitantly used medication					
NSAIDs	148 (0.3%)	1725 (3.3%)	<0.001	1.38 (1.26–1.63)	0.006
Opioid	26 (0.00%)	954 (1.8%)	<0.001	0.25 (0.16–0.38)	<0.001
PPI	135 (0.3%)	994 (1.9%)	<0.001	1.46 (1.13–1.87)	0.003
H2RA	83 (0.2%)	1390 (2.7%)	N/A	N/A	N/A
Anticancer	69 (0.1%)	946 (1.8%)	<0.001	N/A	N/A
AntiTB	90 (0.2%)	659 (1.3%)	<0.001	1.36 (1.01–1.83)	0.041
Antibiotic Types					
Penicillin	406 (0.8%)	7217 (13.7%)	N/A	N/A	N/A
Cephalosporin	902 (1.7%)	17,282 (32.9%)	0.024	N/A	N/A
Monobactam	5 (0.0%)	40 (0.1%)	N/A	N/A	N/A
Carbapenem	188 (0.4%)	3364 (6.4%)	N/A	N/A	N/A
Glycopeptide	3502 (6.7%)	359 (0.7%)	<0.001	1.98 (1.76–2.23)	<0.001
Macrolide	4205 (8.0%)	130 (0.2%)	<0.001	0.50 (0.42–0.61)	<0.001
Lincomycin	1290 (2.5%)	106 (0.2%)	<0.001	1.46 (1.19–1.80)	<0.001
Tetracycline	362 (0.7%)	5 (0.0%)	0.002	0.27 (0.11–0.66)	0.004
Oxazolidinone	112 (0.2%)	19 (0.0%)	<0.001	2.76 (1.68–4.52)	<0.001
Aminoglycoside	1401 (2.7%)	109 (0.2%)	0.004	1.34 (1.10–1.65)	0.005
Fluoroquinolone	8681 16.5%)	516 (1.0%)	N/A	N/A	N/A
Nitroimidazole	1671 (3.2%)	96 (0.2%)	N/A	N/A	N/A
Sulfamethoxazole/Trimethoprim	312 (0.6%)	66 (0.1%)	<0.001	3.14 (2.37–4.16)	<0.001
Mupirocin	104 (0.2%)	0 (0.0%)	0.996	N/A	N/A
Polymyxin B	40 (0.1%)	9 (0.0%)	<.001	3.65 (1.76–7.58)	<0.001
Fusidic acid	3 (0.0%)	1 (0.0%)	N/A	N/A	N/A

**Table 8:** System Organ Class (SOC)-based ADEs caused by antibiotics prescribed for MDR Gram-negative bacterial infection.

System Organ Class	Non-SAE (N = 17,514)	SAE (N = 965)	Total (N = 18,479)	*p*-Value	ROR
Skin and appendage disorders	6248 (35.67%)	213 (22.07%)	6461 (34.96%)	<0.001	0.51 (0.44–0.60)
Musculoskeletal system disorders	107 (0.61%)	6 (0.62%)	113 (0.61%)	0.421	1.02 (0.45–2.32)
Central and peripheral nervous system disorders	861 (4.92%)	45 (4.66%)	906 (4.90%)	0.725	0.95 (0.70–1.29)
Psychiatric disorders	270 (1.54%)	4 (0.41%)	274 (1.48%)	0.009	0.27 (0.10–0.72)
Gastrointestinal system disorders	6009 (34.31%)	111 (11.50%)	6120 (33.12%)	<0.001	0.25 (0.21–0.31)
Liver and biliary system disorders	714 (4.08%)	141 (14.61%)	855 (4.63%)	<0.001	4.02 (3.32–4.89)
Metabolic and nutritional disorders	73 (0.42%)	12 (1.24%)	85 (0.46%)	<0.001	3.01 (1.63–5.56)
Cardiovascular disorders, general	73 (0.42%)	26 (2.69%)	99 (0.54%)	<0.001	6.62 (4.21–10.40)
Heart rate and rhythm disorders	104 (0.59%)	8 (0.83%)	112 (0.61%)	0.361	1.40 (0.68–2.88)
Vascular (extracardiac) disorders	97 (0.55%)	3 (0.31%)	100 (0.54%)	0.324	0.56 (0.18–1.78)
Respiratory system disorders	300 (1.71%)	50 (5.18%)	350 (1.89%)	<0.001	3.14 (2.31–4.26)
Red blood cell disorders	56 (0.32%)	11 (1.14%)	67 (0.36%)	<0.001	3.60 (1.88–6.89)
White cell and RES disorders	479 (2.73%)	89 (9.22%)	568 (3.07%)	<0.001	3.61 (2.85–4.57)
Platelet, bleeding, and clotting disorders	215 (1.23%)	58 (6.01%)	273 (1.48%)	<0.001	5.14 (3.82–6.93)
Urinary system disorders	206 (1.18%)	46 (4.77%)	252 (1.36%)	<0.001	4.21 (3.03–5.83)
Body as a whole—general disorders	1271 (7.26%)	136 (14.09%)	1407 (7.61%)	<0.001	2.10 (1.73–2.53)
Application site disorders	415 (2.37%)	5 (0.52%)	420 (2.27%)	<0.001	0.22 (0.09–0.52)
Resistance mechanism disorders	16 (0.09%)	1 (0.10%)	17 (0.10%)	0.902	1.14 (0.15–8.57)

**Table 9:** Univariate and multivariate analyses for sensitivity analysis.

Predictors	Univariate Logistic Regression (Number of Cases (%))			Multivariate Logistic Regression Analysis	
	SAEs (n = 975)	Non-SAEs (n = 17,702)	*p*-Value	OR (95% CI)	*p*-Value
Sex					
Male	508 (52.10%)	8437 (47.66%)	0.011	1.18 (1.03–1.35)	0.015
Female	463 (47.49%)	9088 (51.34%)		Reference	
Age					
0–9	37 (3.79%)	1301 (7.35%)	<0.001	0.95 (0.92–0.98)	<0.001
10–19	33 (3.38%)	492 (2.78%)			
20–29	69 (7.08%)	991 (5.60%)			
30–39	121 (12.41%)	1556 (8.79%)			
40–49	139 (14.26%)	1896 (10.71%)			
50–59	162 (16.62%)	3491 (19.72%)			
60–69	186 (19.08%)	3135 (17.71%)			
≥70	214 (21.95%)	4511 (25.48%)			
Number of concurrently used medications					
1	518 (53.13%)	12,054 (68.09%)	<0.05	1.85 (1.64–2.09)	<0.001
2	236 (24.21%)	3735 (21.10%)			
3	147 (15.08%)	981 (5.54%)			
≥4	74 (7.59%)	932 (5.26%)			
Number of used antibacterial					
1	622 (63.79%)	12,864 (72.67%)	<0.001	0.63 (0.53–0.75)	<0.001
2	218 (22.36%)	3451 (19.49%)			
≥3	135 (13.85%)	1387 (7.84%)			
Types of concomitantly used medication					
NSAIDs	58 (5.95%)	726 (4.10%)	<0.001	N/A	N/A
Opioid	10 (1.03%)	450 (2.54%)	<0.001	0.17 (0.09–0.32)	<0.001
PPI	32 (3.28%)	237 (1.34%)	<0.001	N/A	N/A
H2RA	34 (3.49%)	516 (2.91%)	N/A	N/A	N/A
Anticancer	36 (3.69%)	429 (2.42%)	<0.001	N/A	N/A
AntiTB	54 (5.54%)	321 (1.81%)	<0.001	N/A	N/A
Antibiotic Types					
Penicillin	80 (8.21%)	2817 (15.91%)	<0.001	0.51 (0.40–0.65)	<0.001
Cephalosporin	177 (18.15%)	3182 (18.98%)	0.888	N/A	N/A
Carbapenem	148 (15.18%)	2495 (14.09%)	0.344	N/A	N/A
Tetracycline	0 (0.00%)	199 (1.12%)	NA	N/A	N/A
Aminoglycoside	35 (3.59%)	400 (2.26%)	0.008	1.47 (1.03–2.11)	0.017
Fluoroquinolone	460 (47.18%)	8257 (46.64%)	0.744	N/A	N/A
Sulfamethoxazole/Trimethoprim	66 (6.77%)	312 (1.76%)	<0.001	2.64 (1.93–3.61)	<0.001
Polymixin B	9 (0.92%)	40 (0.23%)	<0.001	3.21 (1.50–6.76)	0.003

N/A indicates not applicable.

## Data Availability

The data presented in this study are available on request from the corresponding author and KIDS due to the inclusion of patient information.

## Supplementary Materials

The following supporting information can be downloaded at: https://www.mdpi.com/article/10.3390/microorganisms13010136/s1, Figure S1: Heatmap of Tetracyclines; Figure S2: Heatmap of Lincomycin; Figure S3: Heatmap of Nitroimidazole; Figure S4: Heatmap of Sulfamethoxazole/Trimethoprim; Figure S5: Heatmap of Polymyxin B; Figure S6: Heatmap of Oxazolidinones; Table S1: STROBE Statement—Checklist of items that should be included in reports of cross-sectional studies.
